# Phenotyping suicidal behaviour : what could we learn from digital and experimental studies

**DOI:** 10.1192/j.eurpsy.2025.114

**Published:** 2025-08-26

**Authors:** P. Courtet

**Affiliations:** University, Montpellier, France

## Abstract

**Abstract:**

Digital studies based on the continuous monitoring of patients in their natural environment help to refine the suicidal phenotypes. Studies using ecological momentary assessment revealed the existence of different patterns of suicidal ideation (SI) based on both their severity and variability. Specifically, variable SI may be a frequent pattern of suicidal ideation that appears to be related to some clinical features (social withdrawal, impulsive aggression, suicide attempts), rooted in childhood trauma and serotonergic dysfunction and associated with the reactivity to stressful life events. Some individuals, in response to stress could experience both psychological pain and decision-making impairment in social contexts, leading to suicide risk. Then, the description of the suicidal pattern may help to define clinically and biologically homogeneous groups of at-risk patients.

In this sense, experimental studies where patients are submitted to a social stress task reported that patients who were more sensitive to a social stress, as measured with a higher salivary cortisol response, were less depressed, more impulsive, and made suicide attempts with a higher intent. Investigating the regulation of the immune inflammatory response to the social stress task, we recently reported that suicide attempters and ideators showed less dynamic inflammatory stress responses in comparison to psychiatric controls, and that platelet activation responses to stress were blunted in individuals with suicidal ideation. Last, we will present new data of cardiovascular and emotional responses to the virtual Trier Social Stress Test in women with a history of depression and with or without a history of suicide attempt. Combining digital and experimental studies could help to refine the suicidal phenotypes and reduce the heterogeneity of suicidal behaviors that are led by different processes, in order to develop specific therapeutic approaches for a better suicide prevention.

**Disclosure of Interest:**

None Declared

